# Nuclear mechanoprotection: From tissue atlases as blueprints to distinctive regulation of nuclear lamins

**DOI:** 10.1063/5.0080392

**Published:** 2022-06-15

**Authors:** Mai Wang, Irena Ivanovska, Manasvita Vashisth, Dennis E. Discher

**Affiliations:** Biophysical Engineering Labs, University of Pennsylvania, Philadelphia, Pennsylvania 19104, USA

## Abstract

Two meters of DNA in each of our cells must be protected against many types of damage. Mechanoprotection is increasingly understood to be conferred by the nuclear lamina of intermediate filament proteins, but very different patterns of expression and regulation between different cells and tissues remain a challenge to comprehend and translate into applications. We begin with a tutorial style presentation of “tissue blueprints” of lamin expression including single-cell RNA sequencing in major public datasets. Lamin-A, C profiles appear strikingly similar to those for the mechanosensitive factors Vinculin, Yap1, and Piezo1, whereas datasets for lamin-B1 align with and predict regulation by the cell cycle transcription factor, FOXM1, and further predict poor survival across multiple cancers. Various experiments support the distinction between the lamin types and add mechanistic insight into the mechano-regulation of lamin-A, C by both matrix elasticity and externally imposed tissue strain. Both A- and B-type lamins, nonetheless, protect the nucleus from rupture and damage. Ultimately, for mechanically active tissue constructs and organoids as well as cell therapies, lamin levels require particular attention as they help minimize nuclear damage and defects in a cell cycle.

## INTRODUCTION

**FIG. 1.**
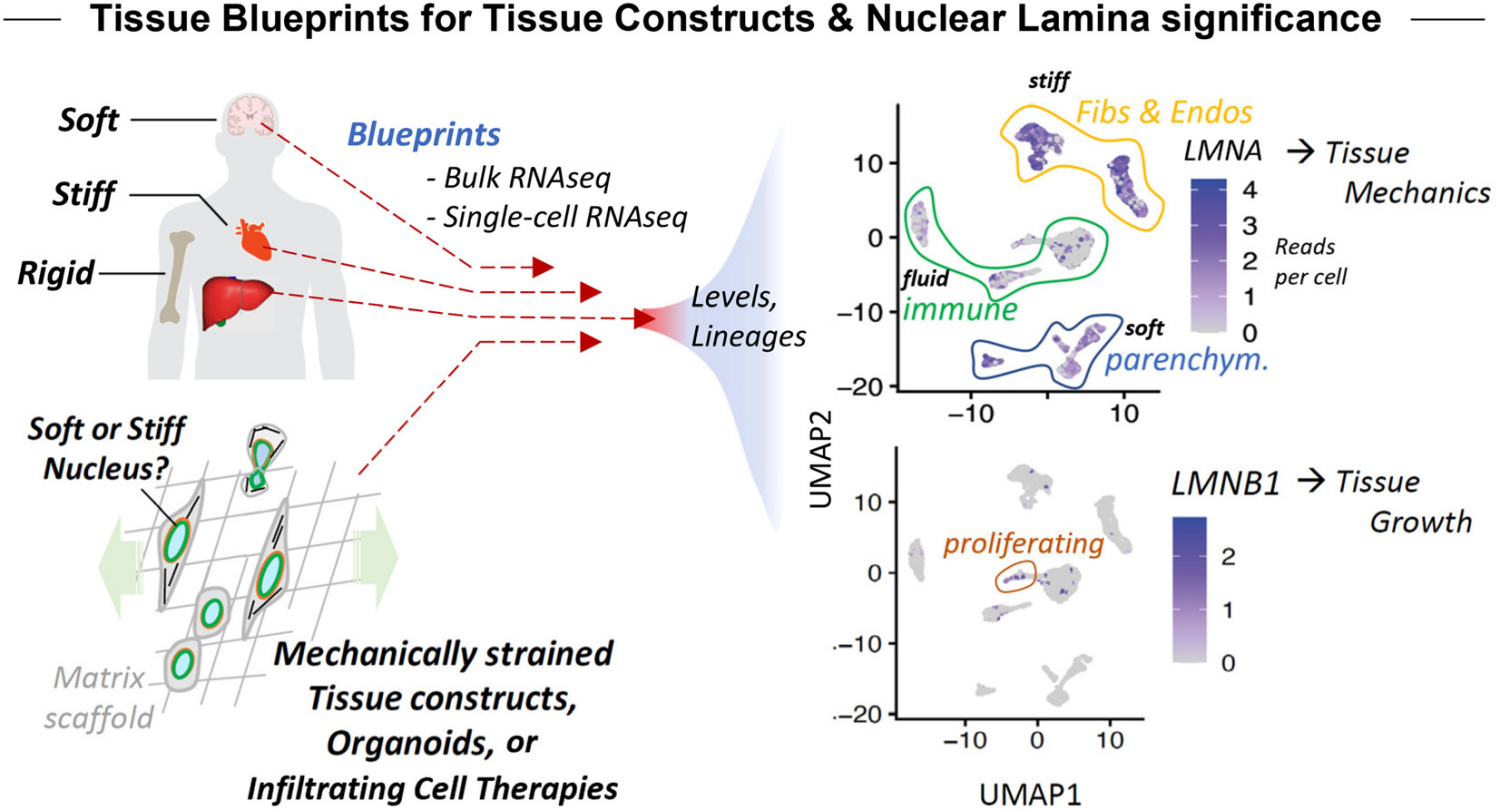
Tissue blueprints for tissue constructs and significance of nuclear lamins. Human tissue atlases can serve, in principle, as “tissue blueprints,” which facilitate the understanding and regenerative engineering of tissue constructs. For example, atlases such as the scatterplots of single-cell RNA-seq data can help illustrate an association between Lamin-A,C expression (*LMNA*) and stiffness and also a distinct relationship of lamin-B1 (*LMNB1*) with cell proliferation. Note “fibs and endos” refers to fibroblasts and endothelial cells, “immune” refers to various types of immune cell lineages, and “parencym.” refers to the tissue-defining cells; the same data are explained in more detail in Fig. 2.

Human tissue atlases are rapidly accumulating gene expression profiles and related data, serving perhaps as blueprints for understanding and constructing tissues (Fig. 1). The atlases are readily accessible, which make them timely and important to assess for possible insight into cells within tissues. Some of the investments are not only expansive and expensive but also disease focused [e.g., >$300M for TCGA: The Cancer Genome Atlas (Ledford, 2015)], which raises possibilities of identifying therapeutic targets. Some of the newest investments provide single-cell resolution, where each datapoint in a cluster (Fig. 1, right plots) represents considerable data from an individual cell—which raises important questions or concerns about what exactly might be learned for cell and molecular biology, biophysics, or bioengineering.

Truth is in the tissues, and the basic question addressed here is “if I have an idea about a role for an interesting molecule in physiology or pathophysiology, how would I start to design a study using publicly available databases to gain insight?” We show how to access standardized datasets and how they can be useful in providing initial insight about associations or correlations between different genes in specific, tissues and cell types. Such genes tend to be upregulated or downregulated together and tend to share similar patterns in the same type of tissues. Thus, when trying to understand the potential role of a certain gene in a process of interest, it is increasingly a good idea to explore available public data for the expression patterns and to compare them with signature genes of the process before pursuing costly and time-consuming experiments.

Nuclear mechanobiology is particularly relevant to probing public datasets, because such data are rich in characterizations of nuclear processes. Key standardized data include gene expression from quantitative RNA-sequencing of bulk tissue and increasingly from single-cell RNA-sequencing (scRNAseq). Epigenetic regulation in a diversity of cultured cell lines is also elaborated in some datasets [e.g., ENCODE: Encyclopedia of DNA Elements (Luo *et al.*, 2020; Rosenbloom *et al.*, 2013)], and biological significance can also be assessed in some datasets (e.g., TCGA) that include patient-specific information on therapy and survival. Questions about data quality and data normalization certainly apply to the big data in the various datasets and atlases, particularly because they are generated by high throughput approaches that focus on breadth rather than depth of insight. Such issues add to the motivations for in-depth investigation of public datasets by cell biologists, biophysicists, and bioengineers who can pursue vigorous follow-up studies. This review aims to illustrate the efforts in two parts: first, an analysis of public atlas data pertinent to nuclear mechanobiology and secondly a review of relevant experiments.

## MINING GENOMIC DATA IN PUBLIC ATLASES FOR LAMIN DIFFERENCES

**FIG. 2.**
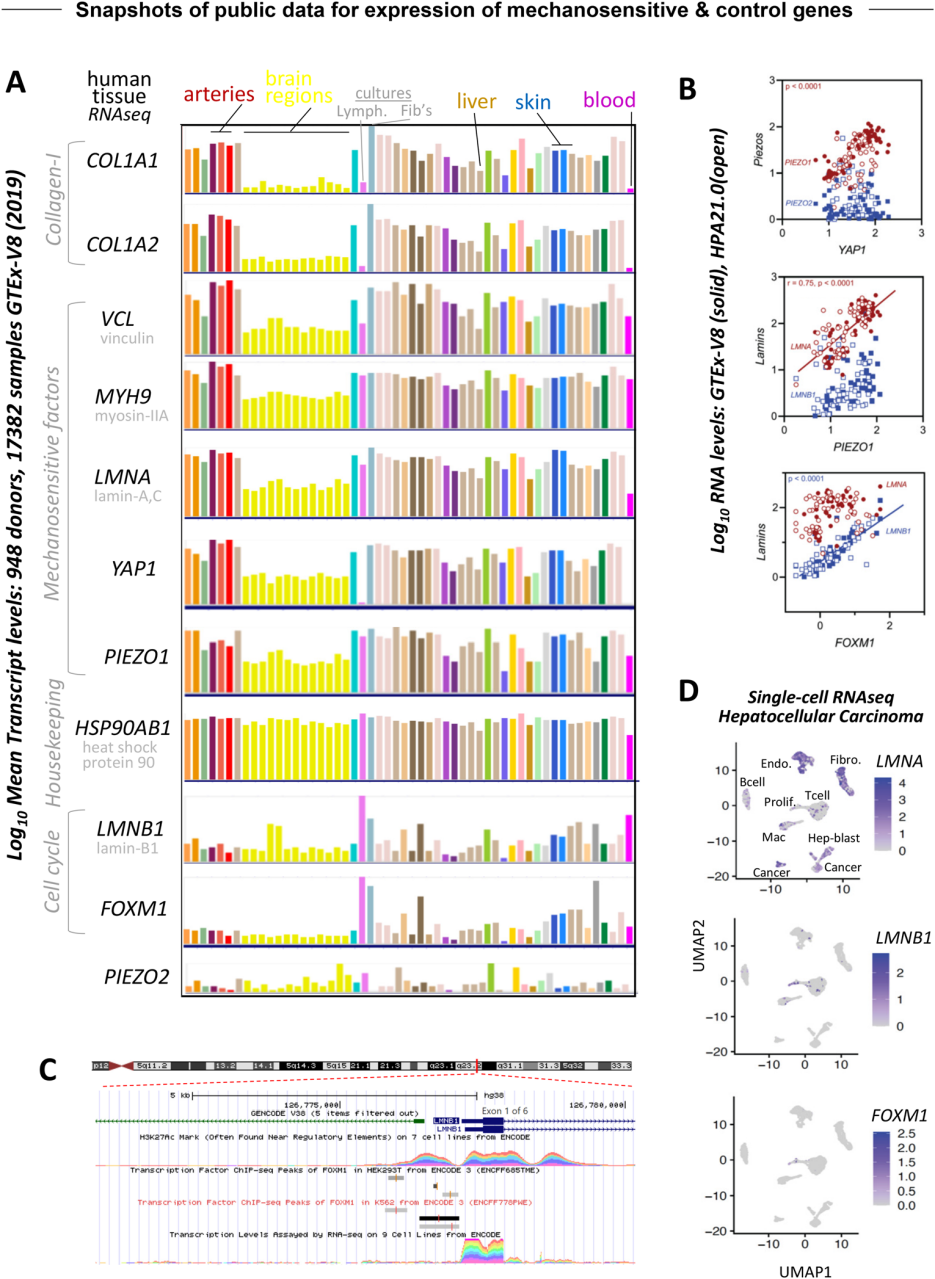
Snapshots of public data for expression of mechanosensitive and control genes. Tissue transcript patterns for collagen-1, the most abundant protein in animals, and for various mechanosensitive factors and related controls. Note that arteries are stiffer than brain, which is soft compared to skin, and of course, blood is fluid. The interested reader is urged to explore the website for details on each tissue shown and for RNA quantitation. Different colors indicate different tissue types from left to right: (adipose—subcutaneous; visceral); adrenal gland; (artery—aorta; coronary; tibial); bladder; (brain—amygdala; anterior cingulate cortex; caudate; cerebellar hemisphere; cerebellum; cortex; frontal cortex; hippocampus; hypothalamus; nucleus accumbens; putamen; spinal cord; substantia nigra); breast—mammary tissue; (cells—EBV-transformed lymphocytes; cultured fibroblasts); (cervix—ectocervix; endocervix); (colon—sigmoid; transverse); (esophagus—gastroesophageal junction; mucosa; muscularis); fallopian tube; (heart—atrial appendage; left ventricle); (kidney—cortex; medulla); liver; lung; minor salivary gland; muscle—skeletal; nerve—tibial; ovary; pancreas; pituitary; prostate; (skin—not sun exposed; sun exposed); small intestine—terminal ileum; spleen; stomach; testis; thyroid; uterus; vagina; whole blood. (a) Scatterplots of bulk tissue RNAseq data from the ENCODE website (GTEx-V8) and from the Human Protein Atlas website (HPA21.0). Each datapoint is the mean for a tissue. (b) Regulation of lamin-B1 gene expression based on ChIP-seq targeting FOXM1 in two cultured cell types with nonzero signal from high (black) to low (light gray). (c) Single-cell RNAseq of liver cancer (Vashisth *et al.*, 2021).

To stimulate use and scrutiny of public data, we begin this review in a tutorial style. We first examine ENCODE data for human tissue expression patterns of mechanosensitive factors. The ENCODE portal www.genome.ucsc.edu provides access to a “Genome Browser” with the latest human dataset “hg38” under “Genomes”; this leads to a browser page with a single-entry box at top into which we enter a specific gene name such as LMNA. Entering this gene name for lamin-A,C re-loads with a cartoon of chromosome-1 (chr1) that shows the location of the *LMNA* gene, and further down the page is a multi-colored bargraph similar to the screenshots of *LMNA* and other mechanosensitive factors [Fig. 2(a)]. The interested reader is urged to enter each gene in the cited website and directly explore and reproduce the data. Our goal is to look for correlations as a basis for causal relationships. Equally important, a lack of correlation suggests little chance of causal relationship.

At the top of our gene set are the two transcripts from RNA-sequencing of many tissues that make collagen-1 protein fibers of the extracellular matrix (*COL1A1*, *COL1A2*); these genes show nearly identical patterns. Recent proteomics of beating embryonic chick heart shows both collagens are rapidly proteolyzed when contractile forces are inhibited (Cho *et al.*, 2019). Whole blood (a fluid tissue) and regions of the brain (a soft solid tissue) all show the lowest average mRNA signal, especially compared to ∼100-fold higher signal in stiffer tissues such as arteries and skin (stiff solid tissues). Dozens of other solid tissues are likewise analyzed by quantitative RNA-seq, but many are difficult to intuit as soft or stiff solid tissues with potentially lower or higher collagen levels. However, two cell culture lines are also included in the profiling: transformed lymphocytes (a fluid tissue cell type) show very low collagen, consistent with the blood profile, and fibroblast cultures (stiff solid tissue cell) show a ∼1000-fold higher signal, consistent with the expected role of such cells in building solid tissues.

Similar patterns of tissue expression are also seen for matrix mechanosensitive factors, but to varying degree: these genes include vinculin (*VCL*) that stabilizes adhesions (Holle *et al.*, 2013; 2016), nonmuscle myosin-IIA (*MYH9*) that typically spans the cytoplasm from nucleus to cell cortex (e.g., Raab *et al.*, 2012), the nuclear intermediate filament gene lamin-A,C (*LMNA*) (Swift *et al.*, 2013; Hadden *et al.*, 2017), and the transcriptional co-activator *YAP1* (Dupont *et al.*, 2011). Also included is the calcium channel *PIEZO1* at the plasma membrane and the endoplasmic reticulum (ER) that is contiguous with the nuclear envelope (Segel *et al.*, 2019; Nava *et al.*, 2020). Distinct patterns of tissue expression are evident for closely related *LMNB1* and *PIEZO2* genes; indeed, all five mechanosensitive genes (*VCL, MYH9, LMNA, YAP1, PIEZO1*) show higher expression in the adherent fibroblast cultures than the non-adherent lymphocytes whereas *LMNB1* and *PIEZO2* are opposite [Fig. 2(a)]. The abundant heat shock gene *HSP90AB1* further shows little variation in tissue or cell line expression, consistent with it being a housekeeping factor. Scatterplots confirm *PIEZO1* correlates well with *YAP1* across diverse tissues and across two RNA datasets [Fig. 2(b)]. This suggests the possibility of a shared regulatory pathway. *LMNA* similarly correlates with *PIEZO1*, whereas *LMNB1* correlates much better with the cell cycle transcription factor *FOXM1*.

In ENCODE, transcription factors and co-factors have also been selectively mapped in their interactions with chromosomal DNA, and data for 338 factors in 130 cell lines constitutes one of the latest large datasets for “Transcription Factor ChIP-seq Peaks.” In this standard method of ChIP-seq, a high-quality antibody immunoprecipitates factor-bound chromatin fragments isolated from nuclei, and then the DNA is sequenced to produce the bound signal as frequency of DNA sequences detected. Modified histones are another common target for ChIP-seq and include Histone-H3 with acetylated-Lys27 (H3K27Ac) that binds near regulatory elements in genes, particularly the promoter region where transcription factors also bind [Fig. 2(c)]. The example shown is the *LMNB1* gene on chromosome-5 with a zoom on exon-1 (of six exons) to show the ChIP-seq signal for H3K27Ac from multiple cell lines. Below this regulated region is the ChIP-seq signal (in gray scale) for FOXM1 protein. Note the convention that nucleic acid is italicized, but protein is not. The ChIP-seq signal is specifically obtained from two cultured cell types, the solid tissue line HEK293T and the blood progenitor K562 line, and the numerical data show for both that the promoter region in *LMNB1* has two or three main binding sites for FOXM1. Such a strong signal is lacking for *LMNA*, which indicates a different type of gene regulation than the cell cycle regulation of *LMNB1*, consistent with the patterns [Figs. 2(a) and 2(b)].

Experiments have recently confirmed FOXM1's cell cycle regulation of *LMNB1* and, thus, provide a mechanistic basis for a gene-gene scaling relationship between *FOXM1* and *LMNB1* across many cancers in TCGA (Vashisth *et al.*, 2021). Furthermore, patients with high levels of *FOXM1* and *LMNB1* have poor survival, consistent with faster cancer growth. Proliferation is afterall a key hallmark of cancer but of course also occurs with stem and progenitor cells in mechanical microenvironments such as soft brain, where *LMNB1* has also been noted as upregulated (Segel *et al.*, 2019).

As with *LMNA*, the *YAP1* gene does not show binding sites for FOXM1 based on the ChIP-seq signal, which again argues against direct cell cycle regulation of *YAP1*. In the pan-cancer analysis of TCGA data (Vashisth *et al.*, 2021), tumors had both higher and lower levels of *YAP1* relative to adjacent normal tissue, and the variation proved similar to other mechanosensitive genes especially *LMNA*. Such results concur with the similarities of expression patterns across normal tissues for *YAP1* and *LMNA* [Figs. 2(a) and 2(b)]. YAP1 might regulate such mechanosensitive genes, but unlike FOXM1, it is not in the ENCODE database of transcription factor ChIP-seq targets. The regulatory basis for the tissue patterns of mechanosensitive gene expression remains important to investigate, but earlier studies provided some insight.

Tissue-level RNA analysis is of course a composite of multiple cell types, but single-cell RNA-seq (scRNAseq) is now becoming publicly accessible for numerous tissues and tumors. We recently analyzed liver cancer data using standard approaches (Vashisth *et al.*, 2021), and the dataset shows more than a half-dozen cell types [Fig. 2(d)]. *LMNA* is expressed in all cell types but is lowest in lymphocytes (T cells, B cells; fluid tissue cell types) and probably highest in fibroblasts (stiff solid tissue cell type), consistent with the cell line results [Fig. 2(a)].

An intriguing corollary to the common finding of low lamin-A,C in fluid tissue cell types is that low lamin-A,C leads to a soft nucleus conducive to 3D migration through small pores (Shin *et al.*, 2013), which is consistent with tissue fluidity. In similar studies of solid tissue stem cells and tumor cells, overexpression of lamin-A,C impeded migration or immobilizes cells (Harada *et al.*, 2014), which would tend to favor tissue solidity, and in strong confinement, a transition to ameboid migration is likewise inhibited by high levels of lamin-A (Lavenus *et al.*, 2022). In contrast, low lamin-A,C facilitates tumor cell invasion and tumor growth (Harada *et al.*, 2014), and recent analyses of TCGA patient data show that *LMNA* is lower than adjacent normal tissues in more than half of tumor types (Vashisth *et al.*, 2021), consistent with prior analyses of protein in lung cancer and breast cancer (Irianto *et al.*, 2016). Such findings are also relevant to cell therapies such as the many engineered Tcell therapies in which these cells are intended to infiltrate tissues, particularly solid tumors.

**FIG. 3.**
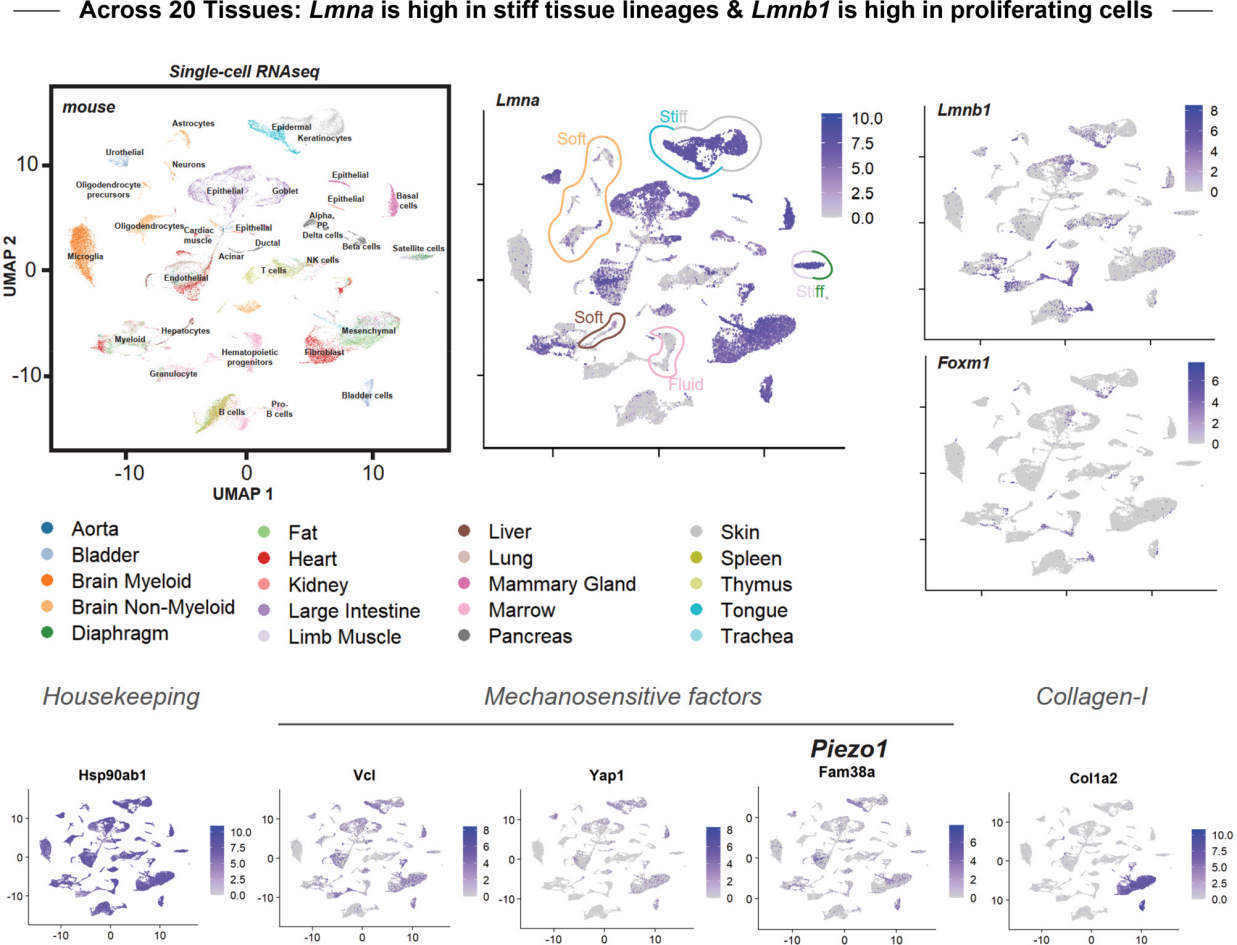
Across 20 mouse tissues: *Lmna* is high in stiff tissue lineages and *Lmnb1* is high in proliferating cells. The UMAP algorithm clusters and disperses lineages more reasonably than the original tSNE analysis (The Tabula Muris Consortium, 2018), but the same color code for tissues and same cluster labels are used here for comparison. The UMAP scatterplot for *Lmna* is similar to that of other mechanosensitive cellular factors, specifically *Vcl*, *Yap1*, and *Piezo1* (*Fam38a*), whereas *Myh9*, *Actb*, and *Hsp90ab1* are more uniformly expressed, and *Col1a2* is restricted to a few lineages. The interested reader can visualize the tSNE versions of such plots by entering genes on the website (https://tabula-muris.ds.czbiohub.org/) and clicking “FACS” for the Method and “ALL” for the Tissue type.

In contrast, *LMNB1* and *FOXM1* are both most readily detected by scRNAseq in proliferating cells [Fig. 2(d)]. These results are consistent with the correlated expression of *LMNB1* and *FOXM1* [Fig. 2(b)] and the underlying regulation [Fig. 2(c)]. Although the sensitivity of scRNAseq and methods of normalization are among the many issues that require deeper study, the above results for one human tissue align reasonably well with an analysis of public data (The Tabula Muris Consortium, 2018) for 20 mouse organs (Fig. 3). In particular, the parenchymal cells that define the specialization of the different tissues tend to show the highest *Lmna* in stiff tissues and lower expression in soft and fluid tissues. (Note that *Lmna* denotes mouse, whereas *LMNA* is the convention for human.) In contrast, *Lmnb1* and *Foxm1* exhibit very similar expression patterns with the latter being a difficult to detect the transcription factor. The distinct trends of *LMNB1* again raise questions of mechanism for how factors such as lamin-A,C are mechano-regulated.

## PROTEIN FOCUSED, MECHANISM EXPERIMENTS ALIGN WITH GENOMIC DATA

**FIG. 4.**
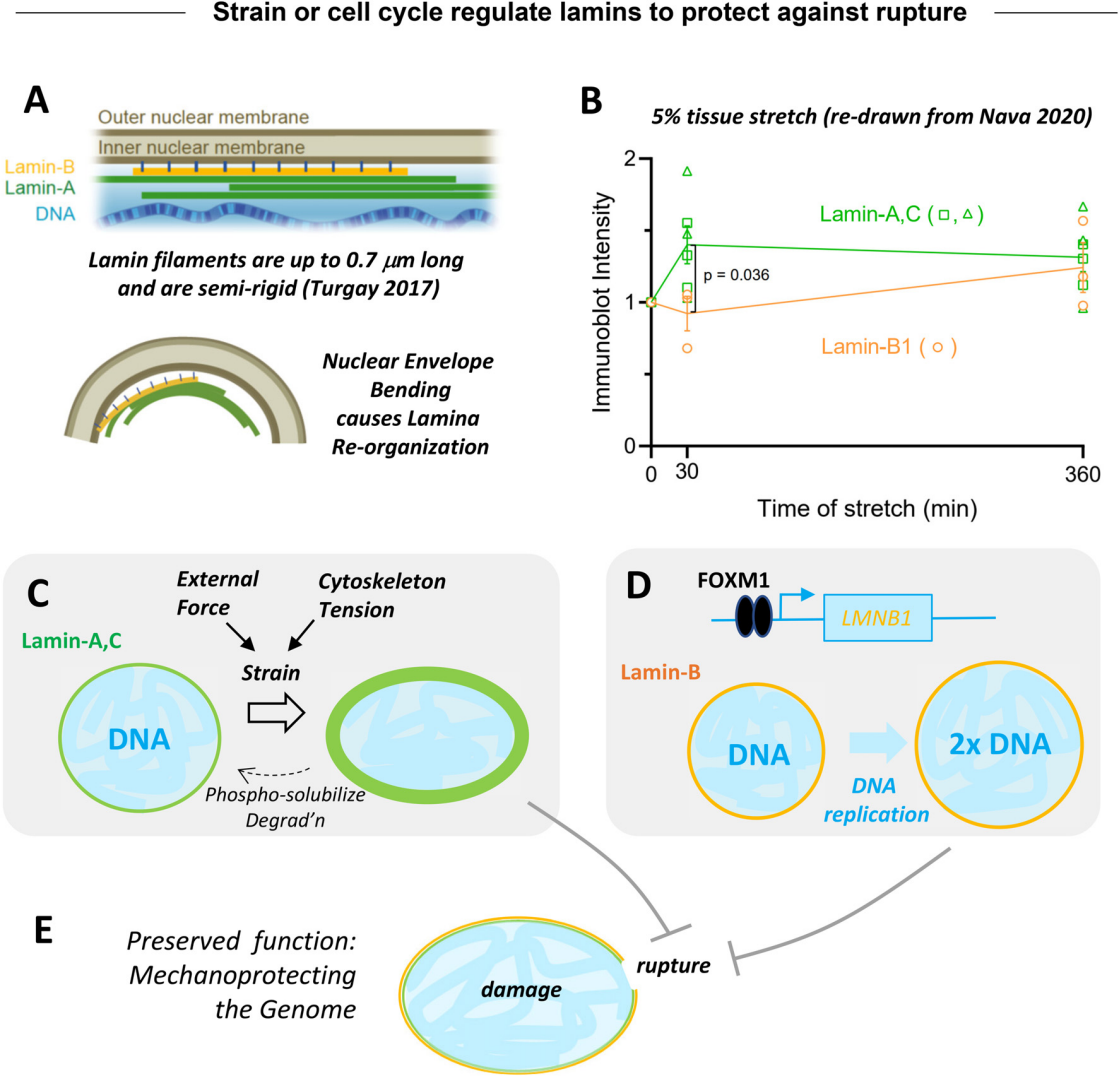
Strain or cell cycle regulate lamins to protect against rupture. (a) Lamin-B has a lipid modification that attaches it to the membrane, so that nuclear bending tends to disrupt its local binding. Lamin-A,C has less direct membrane interactions. At high curvature, lamin-B and lamin-A,C dissociate from the membrane at differing rate. (b) Stretching an epithelial monolayer by 5% for 30 min increases lamin-A,C but not lamin-B1, according to the cited data. At longer time, increased lamin-B1 could reflect cell cycle. (c) Straining the nucleus drives lamin-A,C accumulation with degradation caused by relaxation. (d) Lamin-B1 regulation by cell cycle: more DNA requires more lamina protection. (e) High levels of both lamins suppress rupture of strained nuclei.

Mechanosensing by lamin-A,C was discovered in a proteomics study of diverse adult mouse tissue type (Swift *et al.*, 2013). Specifically, lamin-A,C protein increased as a power law function of tissue stiffness, as did the abundant fibrillar collagens. Lamin-B1 protein (and B2) were relatively constant in comparison. Both lamin types polymerize into filaments that assemble differentially at the nuclear membrane and contribute to nuclear mechanics [Fig. 4(a)] (Turgay *et al.*, 2017); they are in some ways complementary to the fibrillar collagen polymers that are outside. Collagens contribute to bulk tissue mechanics—based on the fact that collagenase fluidizes most solid tissues in minutes. In general, such biopolymers can be expected from basic theoretical principles to exhibit power law behavior, consistent with power law scaling trends across tissues.

Between the nucleus and the extracellular matrix collagens are the cytoskeleton and the adhesion structures of a cell. Lamin-A,C protein levels are affected when perturbing these various structures, at least for primary human mesenchymal stem cells (MSCs) (Swift *et al.*, 2013; Buxboim *et al.*, 2014). On soft matrix that mimics low collagen tissues, cells remain round and have minimal adhesions and low levels of lamin-A,C relative to well-spread cells on stiff or rigid matrices. Inhibition of myosin-II stress in well-spread cells on stiff or rigid matrices again leads to cell rounding and decreased levels of lamin-A,C. In addition, MSCs in standard culture express high lamin-A,C, whereas HSCPs (hematopoietic stem/progenitor cells) that are non-adherent have low lamin-A,C protein.

Mechanoregulation of lamin-A,C at the protein level has been studied with phosphorylation and solubilization from the lamina increasing under soft matrix and low stress conditions (Swift *et al.*, 2013; Buxboim *et al.*, 2014). Interphase cells generally show low levels of lamin-A,C phosphorylation and solubilization (Kochin *et al.*, 2014). Subsequent to phosphorylation and solubilization, lamin-A,C is degraded, and degradation is done by matrix metalloproteases (MMPs) in the nucleus of some cell types (Cho *et al.*, 2019). In other words, the same type of protease responsible for collagen turnover is also responsible for degradation of excess lamin-A,C. This finding can help explain similar expression profiles for collagen-1 and lamin-A,C [Fig. 2(a)]. Indeed, *LMNA* transcription is under feedback control of its protein product (Swift *et al.*, 2013). The end-result of this regulatory circuit seems to be that the levels of lamin-A,C protein are optimized to compliance match the nucleus with the stiffness of the tissue.

*In vivo*, stiff tissues, such as arteries and skin, tend to be mechanically stressed much more than very soft tissues such as brain. In culture, matrix stiffness also promotes cytoskeletal stress (Engler *et al.*, 2006) as well as higher lamin-A,C. Externally imposed strains or stresses might, therefore, mechanoregulate lamin-A,C levels. Monolayers of skin epithelial stem/progenitor cells (EPCs) on stiff substrates were subjected recently to a uniaxial stretch of 5% (Nava *et al.*, 2020), and at 30 min, immunoblots for lamin-A,C and lamin-B's were made and quantified. Pooling all of the results indicates a ∼40% increase in lamin-A,C protein levels at 30 min [Fig. 4(b)], and all of the replicate measurements were greater than or equal to controls. At a much higher stretch of 40% for 30 min, phosphorylation of lamin-A,C was detectably downregulated. Lamin-B1 showed no significant change, consistent with a lack of a stretch effect on mitotic counts (albeit noisy). The larger stretch did not affect lamin-A,C but did associate with increased DNA damage when combined with conditions that suppress heterochromatin. The latter response depended on Piezo1 but not Piezo2, perhaps consistent with the mechanosensitive expression profiles of Piezo1 but not Piezo2 [Fig. 2(a)]. Interestingly, Piezo1 in the endoplasmic reticulum (ER) was key to the stretch sensing, and because the ER is contiguous with the nuclear envelope, stretching of the nucleus can, in principle, affect the ER directly. Whether intact skin experiences 5% stretch more often than 40% stretch is unclear, but these results with externally imposed strain seem consistent with the interphase mechanosensing of matrix stiffness.

Several studies of tissues provide *in vivo* evidence of lamin-A,C mechanosensing. In particular, macrophages isolated from diverse mouse tissues or a tumor plus various tissues have been profiled by RNA-seq in two separate studies (Lavin *et al.*, 2014; Alvey *et al.*, 2017) with nearly identical results. The ratio of (*Lmna*/*Lmnb*) expression reproducibly increases with tissue stiffness from a ratio <1 for bone marrow (which is very soft) to >10 for lung (stiffer). A separate proteomics study was done on living chick embryo heart, which is the first organ to form and which stiffens as it develops day-by-day from an embryo that is about as soft as mature brain. Collagen-1 increases more than any other protein, and lamin-A,C increases almost as much, whereas lamin-B's do not change much at all (Cho *et al.*, 2019). Furthermore, collagenase and myosin-II inhibitors soften the early heart in minutes and quickly decrease levels of collagen, lamin-A,C, and also vinculin—consistent with trends in tissue transcript profiles for human [Fig. 2(a)].

Mechanosensitive regulation of *LMNA* transcription continues to be studied with evidence of feedback from protein levels (Swift *et al.*, 2013; Ivanovska *et al.*, 2017). The SRF pathway certainly deserves more especially, especially since the vinculin gene *VCL* is a definitive target of SRF (Hadden *et al.*, 2017; Costa *et al.*, 2012). Post-transcriptional regulation of *LMNA* mRNA has been documented with suppression in brain tissue by the microRNA miR-9 (Jung *et al.*, 2012). ENCODE shows miR-9 to be very high in brain but largely undetectable in other tissues. In addition, lamin-C is a post-transcriptional spliceform of lamin-A,C that clearly increases with stiffness at the protein level (Swift *et al.*, 2013), but lamin-C is not targeted by miR-9. As mechanoregulation of lamin-A,C transcription pathways and protein levels continue to be explored, additional efforts are focused on the biological importance of lamin-A,C levels.

## MECHANOPROTECTION FOR GENOME INTEGRITY

Defects or deficiencies in lamin-A,C have been linked in many previous studies to DNA damage and impaired proliferation, consistent with broad understanding of DNA damage checkpoints on cell cycle progression. In mechanobiological contexts, however, studies are few that have made similar associations. Studies of embryonic chick hearts treated with myosin-II inhibitors (which suppressed lamin-A,C) showed that washout of the drugs led to rapid increases in DNA damage as contractile stresses recovered in minutes and lamin-A,C levels remained low for hours (Cho *et al.*, 2019). Nuclear rupture was evident at the early timepoints but not later with evidence of rupture including nuclear loss of DNA repair factors that normally minimize DNA damage. Such findings align well with past cell culture studies that showed defects or deficiencies in lamin-A,C, greatly increasing the frequency of nuclear rupture in cells such as fibroblasts with high actomyosin contractility, particularly when they adhere and spread on stiff substrates (Tamiello *et al.*, 2013; Xia *et al.*, 2018).

Defects or deficiencies in lamin-B1 likewise exhibit nuclear rupture *in vivo*, particularly in neurons (Chen *et al.*, 2019). Neurons depend on lamin-B's for any nuclear protection, because lamin-A,C protein levels are very low in soft brain (Swift *et al.*, 2013)—consistent with transcript profiles [Fig. 2(a)]. Mechanisms of DNA damage associated with nuclear rupture continue to be investigated and debated, but a key and expected outcome of increased DNA damage is suppression of cell cycle progression (Xia *et al.*, 2019; Cho *et al.*, 2019). This can help explain why lamin-B1 knockout mice fail to develop the outer cortex of the brain (Chen *et al.*, 2019). Thus, despite the mechanosensitive regulation of lamin-A,C levels being distinct from the direct cell cycle regulation (by FOXM1) of lamin-B1 [Figs. 4(c)–4(e)], the current evidence is that both components of the nuclear lamina mechanoprotect nuclear integrity against DNA damage and cell cycle disruption. Finally, nuclear rupture with effects on cell cycle and cell function is likely relevant to cell therapies with studies of muscle stem cells and mesenchymal stem cells specifically showing that constricted migration modulates stem cell differentiation both in *in vitro* and *in vivo* (Smith *et al.*, 2019).

## WHAT TO LOOK OUT FOR IN PERFORMING SIMILAR STUDIES: PROBLEMS, LIMITATIONS, CHECKS, AND MORE

This review has been written from the perspective of a laboratory with several decades of experimental efforts in mechanobiology, including a focus on the nucleus and its lamins, and so we have an increasingly clear sense of what to look out for in analyzing public datasets—especially from the latest, high resolution omics method of single-cell RNA-seq [per Figs. 1, 2(d), and 3]. At least five issues merit attention with single-cell RNA-seq datasets. First, the dataset should probably be from a single methodology with minimal batch effects. For example, the mouse study analyzed here (Fig. 3) used two cell capturing methods when preparing the libraries: single cells were separated either using fluorescence-activated cell sorting (FACS) or using microfluidic droplets. The FACS method captured a larger diversity of cell types and gave more sequencing reads per cell and more genes per cell. Data generated from the two methods should probably not be mixed, and it would be interesting if unrewarding exercise to try to normalize away the variations that arise from the different technologies.

Second, conclusions drawn from the types of UMAP analyses shown here should focus on the clusters with high cell counts (≫10). Because of the low abundance of transcripts from any one cell, single-cell RNA-seq data show significant cell-to-cell variation that can confound genuine biological heterogeneity with technical shortcomings (Hafemeister and Satija, 2019). A conclusion drawn from the same pattern across many cells will be more reliable and accurate, especially if and when methods emerge for calculating ‘p-values’ on the significance between any two UMAPs or the underlying datasets.

Third, it should be checked that abundant housekeeping genes, such as HSP90AB1, should be expressed in the vast majority of cells in all clusters. Likewise, other such genes, including Actin Beta (ACTB) and Glyceraldehyde-3-Phosphate Dehydrogenase (GAPDH) (Curis *et al.*, 2019; Nikishin *et al.*, 2018), should be checked and included as good controls. Fourth, RNA abundance and undetectable “zeros” are illustrated by differences between LMNA and LMNB1 [Figs. 1, 2(d), and 3] with the latter illustrating how a gene that is known to be highest in late cell cycle (Vashisth *et al.*, 2021) is also often too low to be detected but likely non-zero in many cells in early cell cycle (G1 or G0). Other cell cycle genes, such as FOXM1 and TOP2A, are truly suppressed and zero in early cell cycle, and so “zero” reads in a given cell might simply be a mistake, which underscores the importance of focusing on trends within clusters or parts of a cluster. Finally, if a dataset shows an understandable, non-trivial pattern for several genes of interest across multiple lineage clusters, but the same dataset seems odd for other gene sets that are expected to be similar, then we recommend a conservative interpretation for designing experiments. Support might be found in some comparisons to bulk expression profiles [Fig. 2(a)], but the single-cell datasets are currently sparse (despite their size) and the fact that methods continue to improve suggests a need to improve.

## FUTURE OUTLOOK: TISSUE BLUEPRINTS AND NUCLEAR MECHANOBIOLOGY

Tissue atlases for human, mouse, and many other species are rapidly accumulating bulk and single-cell expression datasets that can serve, in principle, as “tissue blueprints” (Fig. 1). Efforts in such engineering—particularly for mechanically active tissue constructs such as a bioengineered heart but even softer solid tissues such as liver exposed to diverse flows—might employ inert or degradable biomaterials or might be based on organoids among other strategies. Regardless, well-constructed tissue should eventually align in its expression profile with the atlas datasets for the intended tissue. Open access to such public data facilitates such comparisons as do user-friendly interfaces and datasets for diseased tissues such as cancer. It is particularly important that engineered tissue systems and organoids be compared to normal and diseased tissue states with attention to suitably normalized levels of expression. The biological significance of particular expression levels is reasonably well illustrated by lamin-A,C and lamin-B1, with low levels of either relating to nuclear mechanoprotection, nuclear damage, and cell cycle.

## Data Availability

The data that support the findings of this study are available within the article.

## References

[1] Alvey, C. M. , Spinler, K. R. , Irianto, J. , Pfeifer, C. R. , Hayes, B. , Xia, Y. , Sangkyun C. , Dave Dingal, P. C. P. , Jake H. , Lucas S. , Manu T. , and Discher, D. E. , “ SIRPA-inhibited, marrow-derived macrophages engorge, accumulate, and differentiate in antibody-targeted regression of solid tumors,” Curr. Biol. 27(14), 2065–2077 (2017). 10.1016/j.cub.2017.06.005 28669759 PMC5846676

[2] Buxboim, A. , Swift, J. , Irianto, J. , Spinler, K. R. , Dingal, P. C. D. P. , Athirasala, A. , Kao, Y.-R. C. , Sangkyun, C. , Takamasa, H. , Shin, J.-W. , and Discher, D. E. , “ Matrix elasticity regulates lamin-A,C phosphorylation and turnover with feedback to actomyosin,” Curr. Biol. 24(16), 1909–1917 (2014). 10.1016/j.cub.2014.07.001 25127216 PMC4373646

[3] Chen, N. Y. , Yang, Y. , Weston, T. A. , Belling, J. N. , Heizer, P. , Tu, Y. , Kim, P. , Edillo, L. , Jonas, S. J. , Weiss, P. S. , Fong, L. G. , and Young, S. G. , “ An absence of lamin B1 in migrating neurons causes nuclear membrane ruptures and cell death,” Proc. Natl. Acad. Sci. U. S. A. 116(51), 25870–25879 (2019). 10.1073/pnas.1917225116 31796586 PMC6926041

[4] Cho, S. , Vashisth, M. , Abbas, A. , Majkut, S. , Vogel, K. , Xia, Y. , Ivanovska, I. L. , Irianto, J. , Tewari, M. , Zhu, K. , Tichy, E. D. , Mourkioti, F. , Tang, H.-Y. , Greenberg, R. A. , Prosser, B. L. , and Discher, D. E. , “ Mechanosensing by the lamina protects against nuclear rupture, DNA damage, and cell-cycle arrest,” Dev. Cell 49(6), 920–935 (2019). 10.1016/j.devcel.2019.04.020 31105008 PMC6581604

[5] Costa, P. , Almeida, F. V. M. , and Connelly, J. T. , “ Biophysical signals controlling cell fate decisions: How do stem cells really feel?,” Int. J. Biochem. Cell Biol. 44(12), 2233–2237 (2012). 10.1016/j.biocel.2012.09.003 22982240

[6] Curis, E. , Nepost, C. , Laroche, D. G. , Courtin, C. , Laplanche, J.-L. , Etain, B. , and Marie-Claire, C. , “ Selecting reference genes in RT-qPCR based on equivalence tests: A network based approach,” Sci. Rep. 9, 16231 (2019). 10.1038/s41598-019-52217-2 31700128 PMC6838083

[7] Dupont, S. , Morsut, L. , Aragona, M. , Enzo, E. , Giulitti, S. , Cordenonsi, M. , Zanconato, F. , Le Digabel, J. , Forcato, M. , Bicciato, S. , Elvassore, N. , and Piccolo, S. , “ Role of YAP/TAZ in mechanotransduction,” Nature 474(7350), 179–183 (2011). 10.1038/nature10137 21654799

[8] Engler, A. J. , Sen, S. , Sweeney, H. L. , and Discher, D. E. , “ Matrix elasticity directs stem cell lineage specification,” Cell 126, 677–689 (2006). 10.1016/j.cell.2006.06.044 16923388

[9] Hadden, W. J. , Young, J. L. , Holle, A. W. , McFetridge, M. L. , Kim, D. Y. , Wijesinghe, P. , Taylor-Weiner, H. , Wen, J. H. , Lee, A. R. , Bieback, K. , Vo, B.-N. , Sampson, D. D. , Kennedy, B. F. , Spatz, J. P. , Engler, A. J. , and Choi, Y. S. , “ Stem cell migration and mechanotransduction on linear stiffness gradient hydrogels,” Proc. Natl. Acad. Sci. U. S. A. 114(22), 5647–5652 (2017). 10.1073/pnas.1618239114 28507138 PMC5465928

[10] Hafemeister, C. , and Satija, R. , “ Normalization and variance stabilization of single-cell RNA-seq data using regularized negative binomial regression,” Genome Biol. 20, 296 (2019). 10.1186/s13059-019-1874-1 31870423 PMC6927181

[11] Harada, T. , Swift, J. , Irianto, J. , Shin, J. W. , Spinler, K. R. , Athirasala, A. , Diegmiller, R. , Dingal, P. C. , Ivanovska, I. L. , and Discher, D. E. , “ Nuclear lamin stiffness is a barrier to 3D migration, but softness can limit survival,” J. Cell Biol. 204(5), 669–682 (2014). 10.1083/jcb.201308029 24567359 PMC3941057

[12] Holle, A. W. , McIntyre, A. J. , Kehe, J. , Wijesekara, P. , Young, J. L. , Vincent, L. G. , and Engler, A. J. , “ High content image analysis of focal adhesion-dependent mechanosensitive stem cell differentiation,” Integr. Biol. 8(10), 1049–1058 (2016). 10.1039/C6IB00076B PMC5079280 27723854

[13] Holle, A. W. , Tang, X. , Vijayraghavan, D. , Vincent, L. G. , Fuhrmann, A. , Choi, Y. S. , Álamo, J. C. , and Engler, A. J. , “ *In situ* mechanotransduction via vinculin regulates stem cell differentiation,” Stem Cells 31(11), 2467–2477 (2013). 10.1002/stem.1490 23897765 PMC3833960

[14] Irianto, J. , Pfeifer, C. R. , Ivanovska, I. L. , Swift, J. , and Discher, D. E. , “ Nuclear lamins in cancer,” Cell Mol. Bioeng. 9, 258–267 (2016). 10.1007/s12195-016-0437-8 27570565 PMC4999255

[15] Ivanovska, I. L. , Swift, J. , Spinler, K. , Dingal, D. , Cho, S. , and Discher, D. E. , “ Cross-linked matrix rigidity and soluble retinoids synergize in nuclear lamina regulation of stem cell differentiation,” Mol. Biol. Cell 28(14), 2010–2022 (2017). 10.1091/mbc.e17-01-0010 28566555 PMC5541850

[16] Jung, H.-J. , Coffinier, C. , Choe, Y. , Beigneux, A. P. , Davies, B. S. J. , Yang, S. H. , Barnes, R. H. , Hong, J. , Sun, T. , Pleasure, S. J. , Young, S. G. , and Fong, L. G. , “ Regulation of prelamin A but not lamin C by miR-9, a brain-specific microRNA,” Proc. Natl. Acad. Sci. U. S. A. 109(7), E423–E431 (2012). 10.1073/pnas.1111780109 22308344 PMC3289373

[17] Kochin, V. , Shimi, T. , Torvaldson, E. , Adam, S. A. , Goldman, A. , Pack, C.-G. , Melo-Cardenas, J. , Imanishi, S. Y. , Goldman, R. D. , and Eriksson, J. E. , “ Interphase phosphorylation of lamin A,” J. Cell Sci. 127, 2683–2696 (2014). 10.1242/jcs.141820 24741066 PMC4058112

[18] Lavenus, S. B. , Vosatka, K. W. , Caruso, A. P. , Ullo, M. F. , Khan, A. , and Logue, J. S. , “ Emerin regulation of nuclear stiffness is required for fast amoeboid migration in confined environments,” J. Cell Sci. 135, jcs259493 (2022). 10.1242/jcs.259493 35362531

[19] Lavin, Y. , Winter, D. , Blecher-Gonen, R. , David, E. , Keren-Shaul, H. , Merad, M. , Jung, S. , and Amit, I. , “ Tissue-resident macrophage enhancer landscapes are shaped by the local microenvironment,” Cell 159(6), 1312–1326 (2014). 10.1016/j.cell.2014.11.018 25480296 PMC4437213

[20] Ledford, H. , “ End of cancer-genome project prompts rethink,” Nature 517(7533), 128–129 (2015). 10.1038/517128a 25567260

[21] Luo, Y. , Hitz, B. C. , Gabdank, I. , Hilton, J. A. , Kagda, M. S. , Lam, B. , Myers, Z. , Sud, P. , Jou, J. , Lin, K. , Baymuradov, U. K. , Graham, K. , Litton, C. , Miyasato, S. R. , Seth Strattan, J. , Jolanki, O. , Lee, J.-W. , Tanaka, F. Y. , Adenekan, P. , O'Neill, E. , and Cherry, J. M. , “ New developments on the encyclopedia of DNA elements (ENCODE) data portal,” Nucl. Acids Res. 48(D1), D882–D889 (2020). 10.1093/nar/gkz1062 31713622 PMC7061942

[22] Nava, M. M. , Miroshnikova, Y. A. , Biggs, L. C. , Whitefield, D. B. , Metge, F. , Boucas, J. , Vihinen, H. , Jokitalo, E. , Li, X. , García Arcos, J. M. , Hoffmann, B. , Merkel, R. , Niessen, C. M. , Noel Dahl, K. , and Wickström, S. A. , “ Heterochromatin-driven nuclear softening protects the genome against mechanical stress-induced damage,” Cell 181(4), 800–817.e22 (2020). 10.1016/j.cell.2020.03.052 32302590 PMC7237863

[23] Nikishin, D. A. , Filatov, M. A. , Kiseleva, M. V. , Bagaeva, T. S. , Konduktorova, V. V. , Khramova, Y. V. , Malinova, I. V. , Komarova, E. V. , and Semenova, M. L. , “ Selection of stable expressed reference genes in native and vitrified/thawed human ovarian tissue for analysis by qRT-PCR and Western blot,” J. Assisted Reprod. Genet. 35, 1851–1860 (2018). 10.1007/s10815-018-1263-9 PMC6150886 30027530

[24] Raab, M. , Swift, J. , Dingal, P. C. D. P. , Shah, P. , Shin, J.-W. , and Discher, D. E. , “ Crawling from soft to stiff matrix polarizes the cytoskeleton and phosphoregulates myosin-II heavy chain,” J. Cell Biol. 199(4), 669–683 (2012). 10.1083/jcb.201205056 23128239 PMC3494847

[25] Rosenbloom, K. R. , Sloan, C. A. , Malladi, V. S. , Dreszer, T. R. , Learned, K. , Kirkup, V. M. , Wong, M. C. , Maddren, M. , Fang, R. , Heitner, S. G. , Lee, B. T. , Barber, G. P. , Harte, R. A. , Diekhans, M. , Long, J. C. , Wilder, S. P. , Zweig, A. S. , Karolchik, D. , Kuhn, R. M. , Haussler, D. , and Kent, W. J. , “ ENCODE data in the UCSC genome browser: Year 5 update,” Nucl. Acids Res. 41, D56–D63 (2013). 10.1093/nar/gks1172 23193274 PMC3531152

[27] Segel, M. , Neumann, B. , Hill, M. F. E. , Weber, I. P. , Viscomi, C. , Zhao, C. , Young, A. , Agley, C. C. , Thompson, A. J. , Gonzalez, G. A. , Sharma, A. , Holmqvist, S. , Rowitch, D. H. , Franze, K. , Franklin, R. J. M. , and Chalut, K. J. , “ Niche stiffness underlies the ageing of central nervous system progenitor cells,” Nature 573(7772), 130–134 (2019). 10.1038/s41586-019-1484-9 31413369 PMC7025879

[28] Shin, J. W. , Spinler, K. R. , Swift, J. , Chasis, J. A. , Mohandas, N. , and Discher, D. E. , “ Lamins regulate cell trafficking and lineage maturation of adult human hematopoietic cells,” Proc. Natl. Acad. Sci. U. S. A. 110(47), 18892–18897 (2013). 10.1073/pnas.1304996110 24191023 PMC3839750

[29] Smith, L. R. , Irianto, J. , Xia, Y. , Pfeifer, C. R. , and Discher, D. E. , “ Constricted migration modulates stem cell differentiation,” Mol. Biol. Cell 30(16), 1985–1999 (2019). 10.1091/mbc.E19-02-0090 31188712 PMC6727770

[30] Swift, J. , Ivanovska, I. L. , Buxboim, A. , Harada, T. , Dingal, P. C. D. P. , Pinter, J. , Pajerowski, J. D. , Spinler, K. R. , Shin, J. W. , Tewari, M. , Rehfeldt, F. , and Discher, D. E. , “ Nuclear lamin-A scales with tissue stiffness and enhances matrix-directed differentiation,” Science 341(6149), 1240104 (2013). 10.1126/science.1240104 23990565 PMC3976548

[31] Tamiello, C. , Kamps, M. A. F. , van den Wijngaard, A. , Verstraeten, V. L. R. M. , Baaijens, F. P. T. , Broers, J. L. V. , and Bouten, C. C. V. , “ Soft substrates normalize nuclear morphology and prevent nuclear rupture in fibroblasts from a laminopathy patient with compound heterozygous LMNA mutations,” Nucleus 4(1), 61–73 (2013). 10.4161/nucl.23388 23324461 PMC3585029

[26] The Tabula Muris Consortium “ Single-cell transcriptomics of 20 mouse organs creates a *Tabula Muris*,” Nature 562, 367–372 (2018). 30283141 10.1038/s41586-018-0590-4 PMC6642641

[32] Turgay, Y. , Eibauer, M. , Goldman, A. E. , Shimi, T. , Khayat, M. , Ben-Harush, K. , Dubrovsky-Gaupp, A. , Sapra, K. T. , Goldman, R. D. , and Medalia, O. , “ The molecular architecture of lamins in somatic cells,” Nature 543(7644), 261–264 (2017). 10.1038/nature21382 28241138 PMC5616216

[33] Vashisth, M. , Cho, S. , Irianto, J. , Xia, Y. , Wang, M. , Hayes, B. , Wieland, D. , Wells, R. , Jafarpour, F. , Liu, A. , and Discher, D. E. , “ Scaling concepts in 'omics: Nuclear lamin-B scales with tumor growth and often predicts poor prognosis, unlike fibrosis,” Proc. Natl. Acad. Sci. U. S. A. 118(48), e2112940118 (2021). 10.1073/pnas.2112940118 34810266 PMC8640833

[34] Xia, Y. , Ivanovska, I. L. , Zhu, K. , Smith, L. , Irianto, J. , Pfeifer, C. R. , Alvey, C. M. , Ji, J. , Liu, D. , Cho, S. , Bennett, R. R. , and Discher, D. E. , “ Nuclear rupture at sites of high curvature compromises retention of DNA repair factors,” J. Cell Biol. 217(11), 3796–3808 (2018). 10.1083/jcb.201711161 30171044 PMC6219729

[35] Xia, Y. , Pfeifer, C. R. , Zhu, K. , Irianto, J. , Liu, D. , Pannell, K. , Chen, E. J. , Dooling, L. J. , Tobin, M. P. , Wang, M. , and Discher, D. E. , “ Rescue of DNA damage after constricted migration reveals a mechano-regulated threshold for cell cycle,” J. Cell Biol. 218(8), 2545–2563 (2019). 10.1083/jcb.201811100 31239284 PMC6683732

